# *CD38* is associated with bonding-relevant cognitions and relationship satisfaction over the first 3 years of marriage

**DOI:** 10.1038/s41598-021-82307-z

**Published:** 2021-02-03

**Authors:** Anastasia Makhanova, James K. McNulty, Lisa A. Eckel, Larissa Nikonova, Jennifer A. Bartz, Elizabeth A. D. Hammock

**Affiliations:** 1 Department of Psychological Science, University of Arkansas, 216 Memorial Hall, Fayetteville, AR 72701 USA; 2 Department of Psychology, Florida State University, 1107 W. Call St, Tallahassee, FL 32306 USA; 3 Department of Psychology, McGill University, 2001 McGill College Ave, Montreal, QC H3A 1G1 Canada

**Keywords:** Psychology, Genetic markers

## Abstract

Although there are numerous benefits to having a satisfying romantic relationship, maintaining high levels of relationship satisfaction is difficult. Many couples experience declines in relationship satisfaction in the early years of marriage, and such declines predict not only relationship dissolution but also poor mental and physical health. Several recent studies indicate that genetic variation on the CD38 gene (*CD38*), at the single nucleotide polymorphism (SNP) rs3796863, is associated with cognitions and behaviors related to pair bonding; we thus leveraged longitudinal data from a sample of newlywed couples (*N* = 139 genotyped individuals; 71 couples) to examine whether rs3796863 is associated with relationship maintenance processes and, in turn, relationship satisfaction in the early years of marriage. Replicating and extending prior research, we found that individuals with the CC genotype (vs. AC/AA) of rs3796863 reported higher levels of gratitude, trust, and forgiveness and that trust mediated the association between rs3796863 and marital satisfaction. Moreover, the benefits conferred to CC individuals lasted over the first 3 years of marriage. To our knowledge, this is the first study to examine the link between variation in *CD38* rs3796863 and marital functioning over time.

## Introduction

Satisfying romantic relationships play a critical role in helping people reach important goals, such as those relevant to career, social life, and health^1–5^. Perhaps most notably, several meta-analyses demonstrate that satisfying relationships are reliable predictors of better mental and physical health^4,5^, an effect that is at least as powerful as other notable predictors of health, such as physical activity and quitting smoking^3^. Although several mechanisms likely explain this association^6^, many appear to stem directly from high levels of relationship satisfaction^7^. But unfortunately, remaining satisfied in a long-term romantic relationship is notoriously difficult. Not only do divorce rates in numerous industrialized countries hover between 30 and 50%^8,9^, relationship satisfaction typically declines even in the relationships that remain intact [e.g.,^10^]. That said, there is substantial variability in the extent to which people remain satisfied over time [e.g.,^11^], with some people remaining quite satisfied. Given the implications of relationship satisfaction for health and well-being, understanding the factors that determine whether people remain satisfied, or become dissatisfied, may have important practical implications.

Critically, many challenges couples face over the course of their marriage are often present from the beginning^12–14^, which is consistent with research suggesting that some marital difficulties stem from stable individual difference factors^15,16^. In the current research, we focused on one genetic source of individual differences—individual variation on the CD38 gene (*CD38*), a gene that has been linked to social cognition and behavior in rodents^17^, and to positive outcomes in human romantic relationships^18,19^. Specifically, we sought to test whether *CD38* is associated with cognitions and perceptions that help couples strengthen their pair-bond during the challenging newlywed period.

Considerable research, primarily with rodents, has revealed important insights into the biological processes underpinning the maintenance of pair-bonds. Beginning with seminal work on monogamous prairie voles^20–22^, several decades of research have illuminated the importance of the oxytocin system in pair-bonding behavior in non-human animals^23–26^. Notably, recent research has built on this work to suggest a role of peripheral oxytocin levels, and exogenous administration of oxytocin, in attraction and pair-bonding processes in humans [^27–32^; c.f.^33^], as well as in more general processes relevant to social relationships such as trust^34^, perception of others’ emotions^35–37^, cooperative communication^38^, and coping with stress^32^.

In addition to suggesting that peripheral oxytocin levels and exogenous oxytocin administration play a role in human social behavior, research also suggests that genetic differences relating to the oxytocin system play a role in human social behavior. For example, variations in the oxytocin receptor gene (*OXTR*), such as on the polymorphism rs53576, are linked to supportive communication with one’s romantic partner^39^, and marital satisfaction in a sample of adults over 50^40^. Notwithstanding these two studies, recent meta-analyses disagree on whether rs53576 is linked to human sociality generally^41–43^, with evidence for a link between *OXTR* variation and bonding within close relationships being particularly inconsistent^43^.

More recently, however, a different gene—*CD38*—has been implicated in the regulation of oxytocin release and social relationship processes^17,44,45^. *CD38* codes for the transmembrane glycoprotein CD38, which has numerous functions throughout the body. Preliminary evidence suggests that one such function is the modulation of oxytocin release^17^. Specifically, *CD38* knockout mice (vs. wild-type mice) demonstrated lower plasma oxytocin levels, which, in turn, led to deficits in social cognition and in maternal behavior^17^. Consistent with the non-human animal work, recent studies in humans also support the link between *CD38* and adaptive cognitive and behavioral interpersonal processes^18,19,46,47^. These studies have primarily focused on a single-nucleotide polymorphism (SNP), rs3796863, of either an adenine (A) or cytosine (C) located within an intron. For example, one study revealed that young adults with the CC genotype perceived more support from their peers than those with the AC/AA genotypes^46^. In another study, young adults with the CC genotype reported reduced feelings of alienation from their peers and lower rates of suicidal ideation than those with the AA genotype^47^.

More directly relevant to the present research, two studies have linked genetic variation in rs3796863 and pair-bonding processes in romantic relationships. One study demonstrated that, compared to those with the AC/AA genotypes, people with the CC genotype, who were in romantic relationships lasting at least 6 months, expressed more gratitude towards their partner, and had more positive responses to their partners’ expressions of gratitude^18^. Gratitude appears to function, at least in part, to maintain intimacy and relationship satisfaction^48^, at least when it occurs in both members of a close relationship^49^, which suggests *CD38* may ultimately facilitate bonding in humans. Indeed, another study demonstrated that this same *CD38* SNP was associated with other relationship maintenance processes. Specifically, in a study using an intensive repeated measurement method to study couples’ daily behavior, Sadikaj and colleagues^19^ showed that those with the CC (vs. AC/AA) genotype reported engaging in more communal behaviors (e.g., “I expressed affection with words or gestures”) during their interactions with their partner. Furthermore, people with the CC genotype (vs. AC/AA genotypes) perceived their partner as more communal during these interactions, experienced less negative affect, felt less insecure, and reported better overall relationship adjustment. Taken together, these studies suggest that rs3796863 may play an important role in promoting pair bonding and intimacy through a variety of relationship maintenance processes.

Nevertheless, it remains unclear whether the link between rs3796863 and specific relationship bonding processes (e.g., gratitude, communal behavior) translates into higher levels of global relationship satisfaction that are independent of those processes themselves, as well as what other relationship maintenance processes may be involved. We leveraged data from a longitudinal study of newlywed couples to examine these issues. Specifically, 71 newlyweds (*N* = 142 people) completed a battery of questionnaires regarding their relationship cognitions, bonding-related problems, non-bonding-related problems, and relationship satisfaction at a baseline assessment (within 3 months of their wedding); both spouses then reported on their marital satisfaction every four months for the next 3 years (see “Methods”). Participants also provided saliva samples from which we obtained DNA (139 individuals were successfully genotyped). Given the aforementioned research, we focused on variation in rs3796863. It is worth mentioning that Algoe and Way^18^ also examined another *CD38* SNP, rs6449182, which demonstrated stronger links to gratitude measures; however, we chose to focus on rs3796863 because it has been the focus of the majority of studies on human close relationships, and, moreover, one study identified limited variability on rs6449182^19^.

In our data analytic strategy, we sought to conceptually replicate and extend the findings linking the CC genotype to gratitude^17^. We predicted that individuals with the CC (vs. AC or AA) genotype would report higher levels of gratitude toward their spouse. Further, given evidence that people with the CC genotype demonstrate other processes that serve to promote bonding^18,46,47^, we investigated the association between rs3796863 and forgiveness^50^, a related but independent relationship maintenance process, as well as trust, a critical downstream implication of such processes^51^. We predicted that CC individuals would report higher trust in their partner, and would be more forgiving of their partner, than their AC or AA counterparts. We also sought to conceptually replicate and extend prior work linking rs3796863 to global relationship adjustment^18^; in line with that research, we predicted that CC individuals would report greater marital satisfaction than their AA/AC counterparts. Here, we also tested whether rs3796863 is associated with discrete processes that should contribute to relationship satisfaction—namely, perceptions of problems in domains related to pair-bonding and intimacy (e.g., showing affection, amount of time spent together, sex, trust, communication). We predicted that CC (vs. AC or AA) individuals would report fewer pair-bonding problems, but not, importantly, fewer problems in other domains (e.g., friends, religion, money management, decision-making). Finally, we investigated whether the variation in rs3796863 predicted longer-term relationship outcomes by examining the trajectories in relationship satisfaction over the first 3 years of marriage. As noted, this is a particularly vulnerable time for couples; as such, this is a rigorous test of the role of *CD38* in relationship maintenance.

Of note, in order to make the best use of this data set, we also conducted a number of exploratory analyses to test associations between rs3796863 and other processes relevant to pair-bonding that were included in the questionnaires that newlywed couples completed at the start of marriage, including attachment insecurity, commitment, perceptions of romantic alternatives, and jealousy. Although we had did not have strong a priori predictions, these exploratory analyses are valuable in at least two ways. First, they provide insight into potential novel associations between rs3796863 and relationship processes not examined in prior studies. Second, even non-significant associations involving these variables may offer insight into the ways that rs3796863 is discriminately associated with relationship functioning.

## Results

We tested our hypotheses using multilevel modeling, which accounts for the non-independence of responses from husbands and wives (see “Methods” for a comprehensive description of our data analytic approach). Some research suggests racial differences in the prevalence of the reference allele contributing to the CC genotype, such that African Americans have a reference allele frequency of 0.3764 compared to approximately 0.7 for individuals of European ancestry^52,53^. Although we did not have access to our participants’ ancestry, participants did self-report racial information. To ensure that any effects we observed were not due to racial differences in CC prevalence rates, we conducted supplemental analyses to examine the moderating role of race/ethnicity, which are reported in the main text, as well as exploratory analyses to examine race differences on any of the dependent variables, which are described in see Supplemental Materials. We thank an anonymous reviewer for this suggestion.

### Effects of rs3796863 on gratitude, trust, forgiveness, martial satisfaction & perceptions of marital problems at the start of marriage

We first examined the associations between rs3796863 and aspects of marital functioning that we predicted based on the prior literature. Results are presented in Table 1. All associations remained significant when controlling for multiple comparisons^54^, and none of these associations were moderated by sex. As can be seen, the data provided strong support for the hypothesis that the rs3796863 CC genotype is associated with bonding-relevant relationship cognitions in newlywed couples. Specifically, as predicted, CC individuals felt more grateful for their partner, reported higher trust in their partner, were more forgiving of their partner, and were more satisfied with their marriages than were AC/AA individuals. All these effects remained significant in analyses that controlled for self-identified race (see Supplemental Materials), and race did not moderate the association between rs3796863 and gratitude (*p* = 0.598), trust (*p* = 0.187), forgiveness (*p* = 0.778), or marital satisfaction (*p* = 0.117). Although rs3796863 was also associated with perceptions of marital problems in domains relevant to pair-bonding and intimacy (e.g., affection, communication), this association was moderated by race, such that rs3796863 was associated with higher problems among Black participants but not among non-Black participants. Given that we only had 18 participants self-identify as Black, and given that the interaction effect fell just below the threshold for statistical significance, we are hesitant to draw strong conclusions about this effect, especially given that other factors correlated with race may explain it.

**Table 1:** Primary analyses: associations between *CD38* rs3796863 and bonding-relevant cognitions.

Dependent variable	Association with rs3796863 (CC = 0; AC/AA = 1)					
	*B*	*SE*	*t*	*df*	*p*	*r*
Gratitude	**− 0.34**	**0.16**	**− 2.11**	**116.12**	**0.037**	**0.19**
Trust	**− 0.44**	**0.17**	**− 2.62**	**122.36**	**0.010**	**0.23**
Forgiveness	**− 0.46**	**0.16**	**− 2.81**	**116.57**	**0.006**	**0.25**
Marital satisfaction	**− 0.32**	**0.14**	**− 2.28**	**87.32**	**0.025**	**0.24**
**Problems with pair-bonding × race**	1.06	0.53	2.01	98.14	0.047	0.20
Black	1.25	0.51	2.47	97.71	0.015	0.24
Non-Black	0.19	0.14	1.27	98.38	0.208	0.13

The dependent variables are standardized. Bolded effects are significant when correcting for multiple comparisons using the Benjamini–Hochberg procedure^54^. None of the effects were moderated by sex. For problems with pair-bonding, the effect appeared to be moderated by participant race so we are not interpreting the simple effect as significant.

In sum, as predicted, variation in *CD38* rs3796863 was associated with gratitude, trust, forgiveness, and relationship satisfaction. These data thus provided strong support for the hypothesis that the rs3796863 CC genotype is associated with bonding-relevant relationship cognitions in newlywed couples.

### Do relationship maintenance processes mediate the effects of rs3796863 on satisfaction?

**Figure 1:**
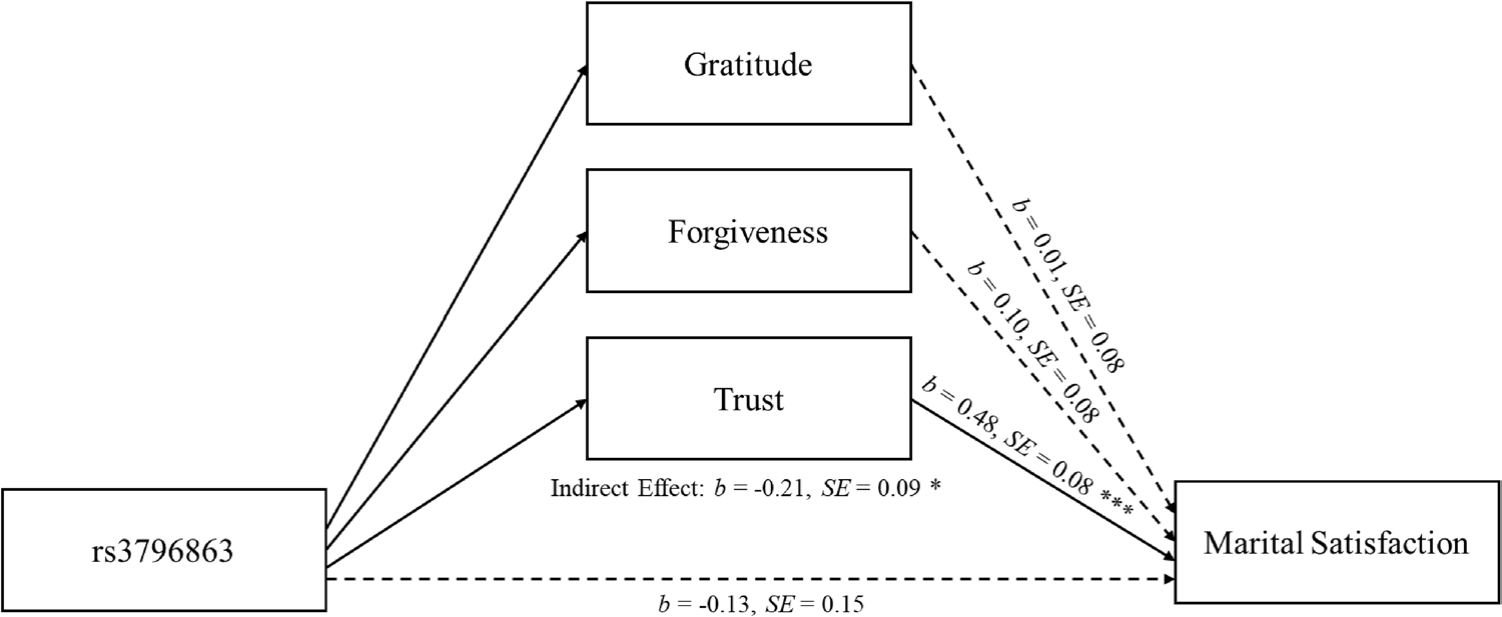
Path b coefficients from the mediation model are shown. Marital satisfaction, gratitude, trust, and forgiveness were standardized. Path a coefficients for the associations between rs3796863 and gratitude, trust, and forgiveness are reported in Table 1. Trust significantly mediated the association between rs3796863 and marital satisfaction. *p < 0.05 ***p < 0.001.

Next, we examined whether the association that emerged between rs3796863 and marital satisfaction did so through its effects on the specific relationship maintenance processes. That is, we tested whether people with the CC genotype were more satisfied with their marriages than those with the AC/AA genotype *because* they are more grateful for, trusting of, and/or forgiving of their partner. To this end, we used the RMediation package^55,56^ to test for these indirect effects. These tests of mediation compute indirect effects based on the coefficients and standard errors of path a and path b. Path a is the association between the predictor and each purported mediator. The coefficients and standard errors for these are reported in the top three rows of Table 1. Path b captures the association between each mediator and marital satisfaction. The coefficients and standard errors for these were estimated in a single new multilevel model that regressed marital satisfaction onto gratitude, trust, and forgiveness simultaneously, while controlling rs3796863 (see Fig. 1). According to these analyses, only trust mediated the effects of rs3796863 on marital satisfaction, *b* = − 0.21, *SE* = 0.09, *p* < 0.05, 95% CI [− 0.39, − 0.05]; controlling trust, the indirect effects of both gratitude, *b* = − 0.005, *SE* = 0.04, 95% CI [− 0.08, 0.07] and forgiveness, *b* = − 0.04, *SE* = 0.04, 95% CI [− 0.14, 0.02], did not reach significance as evidenced by confidence intervals that overlap with 0. These analyses indicate that participants with the CC genotype (compared to those with the AC/AA genotypes) felt more trust toward their partner, which led them to feel more satisfied in their marriages.

### Exploratory analyses on change in marital satisfaction

**Figure 2:**
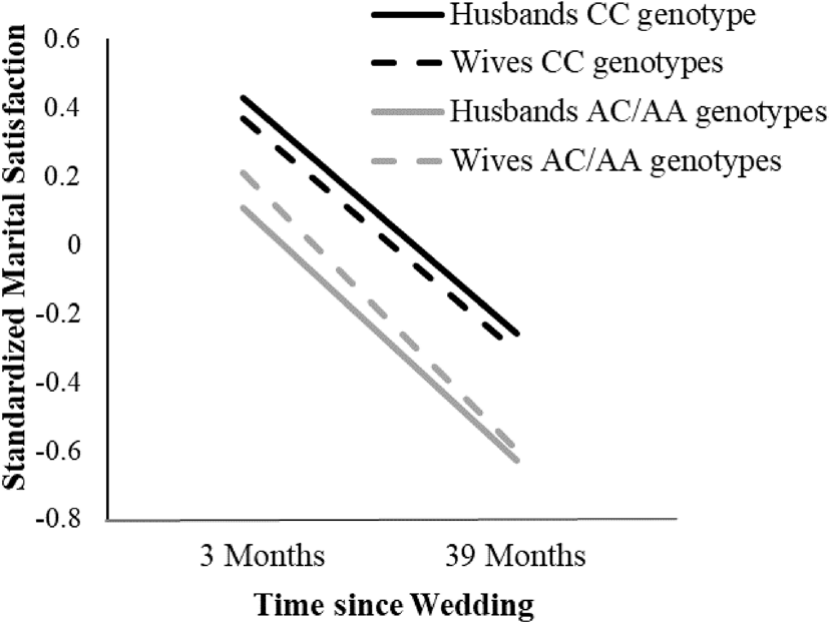
Associations between *CD38* rs3796863 and marital satisfaction over the first 3 years of marriage.

Next, we examined whether the CC individuals remained more satisfied than AC/AA individuals over time by estimating a multilevel growth curve model that included separate fixed and random intercept and time effects for husbands and wives (see Fig. 2). Rs3796863 was not associated with change in relationship satisfaction over time, *b* = − 0.01, *SE* = 0.02, *t*(64) = − 0.72, *p* = 0.473. However, re-centering the time variable such that the intercept represented the end of the study revealed that CC individuals were more satisfied than AC/AA individuals at the end of the study, although this association did not quite reach significance, *b* = − 0.35, *SE* = 0.19, *t*(64) = − 1.86, *p* = 0.068, perhaps due to increased error variance, due to attrition and other factors. In other words, initial differences in marital satisfaction appeared to remain mostly stable over time.

### Exploratory analyses at start of marriage

Several notable findings also emerged in our exploratory analyses (see Table 2). First, compared to AC/AA individuals, CC individuals reported higher levels of commitment. Second, compared to husbands with the AC/AA genotypes, husbands with the CC genotype reported a stronger sense of couple identity and were less likely to notice romantic alternatives during the 14-day daily diary portion of the study. These associations were significantly different from the non-significant effects that emerged among wives. There was no association between rs3796863 and jealousy or attachment insecurity, or several other processes that have been shown to promote relationship functioning. Consequently, although these analyses are exploratory they buttress the hypothesis that rs3796863 may be associated with specific aspects of relationship maintenance rather than general positivity.

**Table 2:** Exploratory analyses: Associations between *CD38* rs3796863 and relationship processes.

Dependent variable	Association with rs3796863 (CC = 0; AC/AA = 1)					
	*b*	*SE*	*t*	*df*	*p*	*r*
General commitment	**− 0.36**	**0.17**	**− 2.08**	**135.00**^**a**^	**0.039**	**0.18**
Primacy of relationship	**− **0.05	0.17	**− **0.29	127.18	0.773	0.03
Satisfaction with sacrifice	0.06	0.17	0.33	128.03	0.741	0.03
Relationship agenda	**− **0.23	0.18	**− **1.29	135.00^a^	0.199	0.11
**Couple identity (CD38 × sex)**	**0.95**	**0.35**	**2.74**	**133.00**	**0.007**	**0.23**
Husbands	**− 0.50**	**0.24**	**− 2.08**	**131.22**	**0.039**	**0.18**
Wives	0.45	0.25	1.83	131.05	0.070	0.16
No desire for alternatives	**− **0.16	0.18	**− **0.94	130.77	0.349	0.08
**Noticing alternatives in diary (CD38 × sex)**	**− 0.78**	**0.31**	**− 2.49**	**132.76**	**0.014**	**0.21**
Husbands	**0.62**	**0.22**	**2.85**	**132.07**	**0.005**	**0.24**
Wives	**− **0.16	0.22	**− **0.73	118.63	0.464	0.07
Problems in other social areas	0.08	0.16	0.52	109.31	0.605	0.05
Problems in non-social areas	0.16	0.15	1.05	106.15	0.298	0.10
All problems	0.21	0.15	1.39	103.94	0.168	0.14
Emotional jealousy	0.21	0.16	1.31	111.36	0.194	0.12
Cognitive jealousy	0.13	0.18	0.76	130.04	0.451	0.07
Behavioral jealousy	0.28	0.18	1.57	135.00^a^	0.118	0.13
Attachment avoidance	**− **0.04	0.18	**− **0.26	127.74	0.799	0.02
Attachment anxiety	**− **0.14	0.17	**− **0.78	127.00	0.436	0.07
Sexual satisfaction	**− **0.28	0.14	**− **1.97	97.90	0.052	0.20
Sexual Frequency	0.09	0.13	0.66	62.01	0.511	0.08
Attributions	0.04	0.18	0.20	132.23	0.843	0.02
Oppositional behavior	0.12	0.15	0.81	98.82	0.420	0.08
Automatic partner attitudes	**− **0.01	0.18	**− **0.04	115.10	0.969	0.004
Automatic attention to alternatives	0.04	0.08	0.49	132.00^a^	0.626	0.04

Bolded effects are significant at α = .05.

^a^Denotes models where a random intercept could not be modeled.

Notably, given the fact that our data also offered insights into potential associations between each gene and partner processes and outcomes, we again tested effects of partner *CD38* on all variables, but no partner effects reached significance (not reported).

## Discussion

We capitalized on data from a longitudinal study of newlywed couples to examine whether variation on the *CD38* SNP rs3796863 was associated with relationship processes and outcomes in newlywed couples. Consistent with our predictions, among both men and women, the CC (vs. AC/AA) genotype was associated with reporting more gratitude toward one’s partner, trust in one’s partner, and forgiveness of one’s partner. Moreover, participants with the CC genotype (vs. AC/AA) reported higher levels of relationship satisfaction, an observation that also replicates prior research^19^. Interestingly, additional analyses revealed that the association between rs3796863 and marital satisfaction was mediated by trust—that is, the reason why those with the CC genotype were more satisfied is because they were more trusting of their partner. This finding is consistent with work suggesting that trust is a key process underlying and explaining the significance of more distal dispositional and cognitive processes that influence romantic relationship dynamics^51^. The trust findings is consistent with prior work linking intranasal oxytocin administration with increased trust^34^, at least among those who were less trusting at baseline^57^. Finally, there was suggestive evidence that CC (vs. AC/AA) continued to be more satisfied after 3-years or marriage, suggesting that the effects of rs3796863 may endure over time, although the fact that this effect did not reach traditional levels of significance indicates it should be treated with caution until replicated.

Our finding that *CD38* is associated with gratitude conceptually replicates and extends findings from several prior studies of romantic couples^18,19^. For example, consistent with Algoe and Way^18^, we found that CC individuals were more likely than AC/AA individuals to report feeling and expressing gratitude toward their partner. Similarly, consistent with Sadikaj and colleagues^19^, we found that CC individuals reported higher levels of marital satisfaction. Importantly, however, our research extends this prior work. First, we show that the effects of the *CD38* SNP extend to other bonding-relevant relationship cognitions (i.e., trust and forgiveness), and we show that it is trust, in fact, that mediates the effect of rs379686 on marital satisfaction. In fact, perhaps the most novel aspect of our work was linking all these processes together, showing that it was specifically the greater levels of trust experienced by those with CC genotype that helped explain why such individuals were more satisfied with their marriages.

Future research may benefit from examining other pathways that may explain the link between the AC/AA genotype and lower relationship satisfaction. Recent research suggests that people with the AC/AA genotype (compared to those with the CC genotype) have increased social sensitivity^58,59^. Specifically, adolescents with the AC/AA genotypes (i.e., carriers of the *A* allele) tracked over the course of 6 years demonstrated higher social anxiety and depression—if they faced high levels of interpersonal stress^58^. Although romantic relationships are associated with numerous psychological and physiological benefits^1–5^, they are also inherently stressful because even the happiest of couples inevitably have problems^60^. Research suggests that when faced with relationship conflict people engage in risk-regulation to manage the competing goals of self-protection and relationship promotion^60^. In such conflict situations, it may be that AC/AA individuals are more likely than CC individuals to shift toward self-protection, thereby lowering their dependence on and intimacy with their partner. In fact, recent research has linked the AC/AA genotype to neuroticism^59^; although this research focused on the link between neuroticism and social anxiety, neuroticism is also reliably linked to lower relationship satisfaction^61^. Consequently, future research may benefit from examining whether variation on rs3796863 mediates the link between neuroticism and marital satisfaction.

Although our understanding of the functional significance of the rs3796863 SNP in humans remains incomplete, research in rodent models indicates that *CD38* influences the synthesis and secretion of oxytocin^17^. Our finding that rs3796863 predicted relationship satisfaction over the course of the newlywed period resonates with other work showing that peripheral oxytocin levels were associated with relationship survival during the perinatal transition period^32^, and facilitated relationship survival in dating couples^31^. We have more to learn about the precise mechanisms by which this polymorphism influences relationship processes. Rs3796863 is located within an intron of the *CD38* gene, meaning that these changes occur in the non-coding region of DNA. They could contribute to regulating the expression of these genes, or simply serve as markers that are closely linked to nearby genetic variants of functional significance. Future work is needed to better understand not only the functional significance of this polymorphism in humans but also the pathway(s) by which it influences relationship maintenance processes.

In contrast to all of our primary hypotheses, which held when correcting for multiple tests, our exploratory analyses involved numerous statistical tests, which can increase the chance of spurious effects. Accordingly, readers should be cautious in interpreting those effects. That said, several of the significant effects that emerged in our exploratory analyses were consistent with the overall pattern of predicted effects. For example, participants with the CC (vs. AC/AA) genotype were more committed to the relationship. Commitment is thought to be, at least partly, a downstream consequence of marital satisfaction; thus the effects of the CC genotype on commitment may come about because of its effects on marital satisfaction and/or other more proximal bonding-related processes (e.g., trust). Interestingly, our exploratory analyses also identified two effects specific to men: husbands with the CC genotype (vs. husbands with the AC/AA genotypes) tended to report having a stronger couple identity and were less interested in romantic alternatives. Couple identity is a facet of commitment that is associated with greater personal dedication to the relationship, specifically the perception that one and one’s partner are a team^62^. Attractive alternatives, on the other hand, are a threat to relationship commitment; paying less attention to and devaluing alternatives is associated with better relationship outcomes and pair-bond maintenance^63^. Although most other effects of rs3796863 were consistent across husbands and wives, these latter findings suggest that rs3796863 may affect men’s commitment through these additional routes. Overall, findings from the primary and exploratory analyses are consistent with the idea that the CC genotype predisposes people to evaluate their relationships more positively and engage in benevolent cognitions that promote intimacy. Notably, people’s attachment anxiety and attachment avoidance were not associated with rs3796863. This may be because attachment is strongly influenced by social learning and caregiver behavior^64^ rather than genetic predispositions; other genetic polymorphisms in the oxytocin system (such as variation on the SNP rs53576 of the oxytocin receptor gene) are similarly not linked to attachment insecurity^65^. Nevertheless, future research may benefit from conceptually replicating the specific effects in both our primary and exploratory analyses.

Several limitations of this work are worth considering. First, our results are limited to a sample of individuals of European ancestry practicing Western cultural norms of romantic relationships. Not only are genetic differences differentially distributed in various ancestries, as noted, but the same genetic differences may be uniquely associated with romantic relationship processes in Western cultures compared to romantic relationships common to other cultures [e.g., arranged marriages^66^]. Future research may benefit from examining how genetic variation on *CD38* may be associated with relationship processes in cross-cultural, more diverse, and larger samples. Additionally, the CC genotype may be differentially associated with relationship processes even within the North American culture. In our sample, Black participants reported more pair-bond related problems and lower marital satisfaction than non-Black participants (see Supplemental Materials). One reason for this association may be due to the fact that members of minority groups face greater sociodemographic stress and are more likely to have lower socioeconomic status (SES) coupled with the fact that SES is an important moderator for the effectiveness of relationship maintenance strategies^67^. It may be that the CC genotype exerts more powerful effects in these more vulnerable groups. The lack of diversity in our sample and the absence of targeted measures precluded us from examining these associations directly in the present data. Future research could examine whether factors such as race, socioeconomic status, and sociodemographic stress affect the link between variation on rs3796863 and perceptions of problems and/or relationship intimacy.

A second limitation is that, because our hypotheses were guided by prior work focusing specifically on rs3796863, we focused on this single polymorphism. Thus, our findings join a growing body of literature suggesting the involvement of *CD38* in relationship processes, but do not preclude the role of other *CD38* polymorphisms, or other genes, in relationship processes. Indeed, other polymorphic sites on *CD38* (e.g., rs6449182) have been linked to relationship dynamics. Relatedly, it is important to acknowledge that there are legitimate concerns regarding the potential for spurious findings in candidate gene studies like this one, including potential issues with spurious findings and the oversimplification of—or even error regarding—the link between a gene and social behavior. Thus, although we had a priori hypotheses about the associations between rs3796863 and the marital processes we assessed, these hypotheses were based in a literature that may suffer from publication bias. To increase transparency of the present research, we have been explicit about the method, predictions, and results of all analyses conducted, and followed STREGA recommendations^68^. To our knowledge, there has not been a genome wide association study (GWAS) on romantic relationship maintenance as a quantitative trait in the GWAS catalog [https://www.ebi.ac.uk/gwas/home; ^69^]. The present research will thus provide valuable phenotypic descriptions for future GWAS on romantic relationship maintenance. Given the associations reported in this paper, and in the prior literature, it would be important to conduct a GWA study examining marital satisfaction, bonding-relevant cognitions (e.g., trust, gratitude, forgiveness), and relationship changes over time.

A final limitation is our small sample size, which was dictated by our original longitudinal study of newlywed couples (a population that is notoriously difficult to recruit). We utilized all physiological samples that were available at the time when funding for the current research question was obtained, leaving us with data from 139 genotyped individuals for our analyses. Because we used a convenience sample to test the current predictions, our findings should be replicated. That said, our genotyped sample was comparable to or larger than those used in past research^18,19^. Overall, we hope these findings encourage more close relationships researchers to collect physiological samples so that data from several longitudinal samples can be pooled together to test future hypotheses.

## Conclusion

Taken together, these findings offer new insights into research at the intersection of genetics and romantic relationships. Regarding relationships, these findings offer novel insights into important precursors that may help or hinder newlyweds as they navigate the first few years of marriage. Polymorphisms on the *CD38* gene were associated with bonding-relevant cognitions (gratitude, trust, and forgiveness), and overall marital satisfaction over the course of the first 3 years of marriage. The reported results represent group-level effects that are relatively small in size and are just one potential source of variance in people’s relationship processes and satisfaction over time. Regarding genetics, these findings support existing findings linking *CD38* and the SNP rs3796863 to romantic relationship processes. Indeed, with the addition of our replication study to the existing literature^18,19^, there are now three independent samples, by three different research teams, that provide consistent evidence linking the CC genotype (compared to the AC/AA genotypes) to positive romantic relationship cognitions and outcomes.

## Method

### Participants

Participants were 142 people (from 71 couples). This sample was a subset of a larger, longitudinal study on newlywed relationships for whom samples for DNA analyses were collected. The larger sample contained a total of 240 members of 120 couples and has been described in prior research [e.g., ^63^]. Saliva samples from the other couples were either depleted prior to the funding that allowed the genetic assays or DNA could not be isolated. Additionally, given that some hypotheses were derived from perspectives of processes guiding heterosexual relationships, data were excluded from one couple in the sample that was not heterosexual. Husbands were on average 32 years old (*M* = 32.20, *SD* = 10.98, range 20–72); wives were on average 30 years old (*M* = 30.33, *SD* = 8.48, range 21–55). For the majority of the couples this was their first marriage (76.1%) and most did not have children (73.9%). The majority of couples were White/Caucasian (73.2%). The study procedures were approved by the Florida State University Institutional Review Board (IRB) and research was conducted in accordance with the relevant guidelines and regulations set forth in the Belmont Report of the National Commission for the Protection of Human Subjects of Biomedical and Behavioral Research in 1979 as well as the American Psychological Association. All participants provided written informed consent to participate in the study.

### Procedure

After telephone-based eligibility screenings, couples were sent either paper packets of questionnaires or an online, individualized link to a Qualtrics survey. Husbands and wives were instructed to complete the questionnaires independently prior to an upcoming lab session, which was scheduled within three months of their wedding. During the lab session, couples engaged in a series of problem-solving discussions and provided saliva samples via passive drool from which DNA was isolated. After the lab session, both couple members completed a brief survey every night for the 14 nights subsequent to the session (i.e., a daily diary). Finally, couple members responded to several of the same questionnaires again every four months for 3 years, including the measures of relationship satisfaction, which we analyzed in exploratory analyses.

## Materials

### Primary dependent measures

#### Gratitude

Participants reported their general disposition of gratitude toward their partner^49,70^. Participants rated the frequency with which they generally felt and expressed gratitude toward their partner (α = 0.82, e.g., I feel appreciation for the things that my partner does for me” on a scale of 1 (Never) to 5 (Frequently). Most participants expressed gratitude frequently (*M* = 4.78, *SD* = 0.34). We predicted that individuals with the CC genotype would report greater gratitude compared to individuals with the AC/AA genotypes.

#### Trust

Participants completed the Trust subscale of the Perceived Relationship Quality Components Inventory^71^ which included three questions (α = 0.86) about their trust toward their partner (e.g., “How much do you trust your partner?”). Participants responded using a 1 (Not at all) to 7 (Completely) scale. Most participants reported high levels of trust (*M* = 6.56, *SD* = 0.74). We predicted that individuals with the CC genotype would report greater trust compared to individuals with the AC/AA genotypes.

#### Forgiveness

Participants’ forgiveness was measured by assessing their likelihood to forgive their partner if he or she committed five hypothetical marital transgressions that varied in severity [e.g., the partner snapped at and insulted the spouse; see^72^]. Participants reported whether they would express forgiveness for each scenario on a scale of 1 (Definitely no) to 7 (Definitely yes). Participants rated themselves to be slightly above the scale midpoint on average (*M* = 4.76, *SD* = 1.23). We predicted that individuals with the CC genotype would show a greater tendency to forgive their partners compared to individuals with the AC/AA genotypes.

#### Perceived severity of problems

Participants also completed the Inventory of Marital Problems^73^. The questionnaire asks participants to indicate on a scale of 1 (Not a Problem) to 11 (Major Problem) the extent to which each of the 19 different common marital problems is an issue for their marriage. Before conducting analyses, the first two authors independently separated the domains into three composites for the primary analyses and reconciled discrepancies. One composite comprised problems relevant to pair-bonding specifically (showing affection, amount of time spent together, recreation and leisure time, sex, trust, jealousy, and communication). The second comprised problems relevant to social interactions outside of the pair-bond (children; in-laws, parents, and relatives; friends). The third comprised problems in non-social areas of the relationship (religion, household management, making decisions, unrealistic expectations, independence, money management, solving problems, drugs and alcohol, career decisions). Participants did not perceive many problems in pair-bonding (*M* = 2.65, *SD* = 1.43), in social interactions outside of the pair-bond (*M* = 2.62, *SD* = 1.46), or in non-social domains (*M* = 2.53, *SD* = 1.45). We predicted that individuals with the CC genotype would report fewer problems relevant to pair-bonding (the first composite) compared to individuals with the AC/AA genotypes; the other domains of problems were included in the exploratory analyses because we had no strong predictions that they would differ based on rs3796863 genotype.

#### Marital satisfaction

Every four months for 3 years, participants completed three measures of relationship satisfaction: the Quality of Marriage Index^74^, the Semantic Differential^75^, and the Kansas Marriage Satisfaction scale^76^. Given all scales are meant to measure the same construct, global marital satisfaction, which is confirmed by the high correlations among the scales (all *r*’s > 0.887), we standardized each scale by creating a Z-score and created a composite of the three measures (which was also standardized). As would be expected and as is reported elsewhere^63^, most newlyweds report being very satisfied with their marriages. We predicted that individuals with the CC genotype would report being more satisfied at the start of marriage compared to individuals with the AC/AA genotypes. We additionally explored whether the trajectory of marital satisfaction over the first 3 years of marriage was associated with variation on rs3796863.

### Exploratory dependent measures

Because the broader study aim was to explore associations between rs3796863 and pair-bonding, the most relevant questionnaires to pair-bond strength and maintenance were chosen from the broader baseline battery. Before conducting analyses, the first two authors independently compiled lists of measures that were then reconciled into a complete list that is presented in this manuscript. No other questionnaires from the battery were examined in relation to rs3796863.

#### Commitment

Participants completed multiple commitment measures. Participants completed the Rusbult commitment scale^77^, which examined general desires toward commitment in their relationship (α = 0.72, *M* = 8.13, *SD* = 0.77, e.g., “I am committed to maintaining my relationship with my partner”). Participants also completed the Commitment Inventory^62^ which consisted of 10 subscales. We selected six subscales that were most relevant to pair-bonding: relationship agenda (α = 0.71, *M* = 6.71, *SD* = 0.56, e.g., “I want this relationship to stay strong no matter what rough times we may encounter”), couple identity (α = 0.76, *M* = 6.14, *SD* = 0.80, e.g., “I like to think of my partner and me more in terms of ‘us’ and ‘we’ than ‘me’ and ‘him/her’”), prioritization of relationship (α = 0.75, *M* = 6.27, *SD* = 0.73, e.g., “My relationship with my partner comes before my relationships with my friends”), satisfaction with sacrifice (α = 0.84, *M* = 5.71, *SD* = 0.97, e.g., “It can be personally fulfilling to give up something for my partner”), availability of partners (α = 0.80, *M* = 3.64, *SD* = 1.19, e.g., “It would be very difficult for me to find a new partner”), and alternative monitoring (α = 0.73, *M* = 5.96, *SD* = 1.02, e.g., “I am not seriously attracted to members of the opposite sex other than my partner”). We omitted the remaining four subscales because they did not seem to pertain to the pair-bond itself but instead captured broader individual differences and contextual variables: social pressure, perceived morality of divorce, structural investments, and meta-commitment. We created a composite (α = 0.74) of the two subscales relevant to alternatives but analyzed the other subscales individually.

#### Jealousy

Participants completed the Multidimensional Jealousy Scale^78^ which consists of three subscales that assess emotional, cognitive, and behavioral jealousy. For the emotional jealousy subscale, participants were asked to indicate how they would react emotionally to the situations described by each statement (*n* = 8, α = 0.87, *M* = 4.50, *SD* = 0.99, e.g., “Your spouse comments to you on how great looking a particular member of the opposite sex is”) on a scale of 1 (Very pleased) to 7 (Very upset). For the cognitive jealousy subscale, participants were asked to indicate how often they had thoughts about their partner described by each statement (*n* = 7, α = 0.87, *M* = 1.70, *SD* = 0.88, e.g., “I suspect that my spouse is secretly seeing someone of the opposite sex”) on a scale of 1 (Never) to 7 (All the time). For the behavioral jealousy subscale, participants were asked to indicate how often they engaged in behaviors described by each statement (*n* = 8, α = 0.82, *M* = 1.85, *SD* = 0.88, e.g., “I look through my spouse’s drawers, handbag, pockets, phone, or emails”) on a scale of 1 (Never) to 7 (All the time).

#### Noticing alternatives on a daily basis

Following the lab session, participants completed a two-week diary. Each day, participants answered a question “To what extent did you notice members of the opposite sex today?” on a scale of 1 (Not at all) to 7 (Very much). We averaged participants’ answers across the diary days they completed (*M* = 2.08, *SD* = 1.36).

#### Attachment Insecurity

Participants reported their levels of attachment insecurity using the Experiences in Close Relationships Scale—Revised [ECR-R^79^]. The ECR-R is comprised of an attachment anxiety subscale (α = 0.92, *M* = 2.22, *SD* = 0.99, e.g., “I am afraid to lose my partner’s love”) and an attachment avoidance substance (α = 0.93, *M* = 2.23, *SD* = 0.92, e.g., “I prefer not to show a partner how I feel deep down”). Participants rated their agreement with 18 statements per subscale on a 1 (Strongly Disagree) to 7 (Strongly Agree) scale.

#### Sexual satisfaction

Participants completed the Index of Sexual Satisfaction^80^, which contains 25 items (α = 0.93, e.g., “I think that our sex is wonderful”) using a scale from 1 (None of the time) to 7 (All of the time).

#### Sexual frequency

We assessed sexual frequency in two ways. In the main questionnaire battery, participants responded to an open-ended question “Approximately how many times have you had sexual intercourse with your spouse over the past 4 months?” Additionally, in the two week diary that followed the initial lab session, participants reported daily whether they had sex with their partner. For analyses using the diary measure, we computed the percentage of each individuals’ diaries completed during which sex was reported within the total number of diaries completed (*M* = 29.91, *SD* = 20.23).

#### Attributions

Participants completed the Relationship Attribution Measure^81^ that assesses people’s attributions of negative behaviors from one’s partner (e.g., one’s partner criticizing the person). Participants responded using a scale from 1 (Not at all) to 7 (Completely) and we averaged responses across scenarios and attributions (*M* = 3.66, *SD* = 1.02).

#### Oppositional behavior

During the laboratory session that occurred within three months of the couples’ wedding, couples engaged in a series of problem-solving discussions that were video recorded. Each individual’s speaking turn was coded for oppositional behavior following an established coding scheme^82^. Oppositional behavior codes comprised 3 direct oppositional behaviors (blaming, rejecting, making demands of the partner) and 5 indirect oppositional behaviors (sarcasm, hostile joking, hostile questioning, mind-reading, denying responsibility). A second coder overlapped on 18% of the conversations (ICC = 0.74). Analyses focused on the proportion of actors’ total speaking turns that received oppositional codes that were averaged across the four conversations (*M* = 0.08, *SD* = 0.10).

#### Automatic partner attitudes

During the lab session, participants completed the Partner Evaluative Priming Task that assessed automatic partner attitudes [for task description, see^83^]. Higher values represented more positive automatic partner attitudes (*M* = 53.43, *SD* = 103.29).

#### Automatic attention to romantic alternatives

During the lab session, participants completed a dot probe attention task that assessed the latencies with which they shifted their attention away from a quadrant on the computer screen in which target faces appeared to categorize a circle or a square in a different quadrant^84^. We examined latencies following the attractive opposite-sex targets (*M* = 506.79, *SD* = 82.73) while controlling latencies following attractive same-sex targets, average same-sex targets, and average opposite-sex targets^63^.

### Genotyping

Saliva samples were collected via passive drool into 20 mL plastic scintillation vials and frozen at − 20 °C immediately after the lab session. At a later time, samples were thawed and centrifuged. After we removed supernatant from each sample for the purpose of conducting hormone assays (not a focus of the current report), we froze the pellets again. Pellets were later thawed for a second time before purification. For most samples, pellets and some supernatant were pooled from two samples. We purified DNA using a protocol adapted from past research^85^. Out of 142 samples that we attempted to purify, we obtained adequate DNA concentrations from 139. We performed control analyses on agarose gel on 6 samples to test for DNA degradation; no issues with DNA integrity were observed. We used a Nanodrop to determine yield (average yield was 494 ng/μL) and 260/280 ratio. If the 260/280 ratio was below 1.80, samples were additionally run through a spin column from a Qiagen DNeasy Blood and Tissue kit following manufacturer instructions. DNA was diluted to 28 ng/mL in TE buffer before the assays.

Genotyping for single nucleotide polymorphisms was conducted using a Taqman SNP Genotyping Assay [rs3796863: C___1216944_10; which reports the nucleotides from the complementary (−) strand of the CD38 gene]. All samples were run in duplicates on the plate with complete concordance. Allele frequency did not differ based on participant sex, χ^2^(2) = 0.841, *p* = 0.66. We followed previous work^18,19^ to code the CC genotype as 0 and AC/AA genotypes both as 1; genotype frequencies are reported in Table 3.

**Table 3:** Genotype counts for rs3796863.

Genotype	Husbands	Wives	Total
**Entire sample (N = 139)**			
C/C	29	24	53
A/C	30	31	61
A/A	11	14	25
**White participants (n = 101)**			
C/C	23	21	44
A/C	22	22	44
A/A	7	6	13
**Black participants (n = 18)**			
C/C	3	0	3
A/C	4	3	7
A/A	3	5	8

Frequencies for each genotype of *CD38* rs3796863 in the total sample, as well as for White and Black participants separately.

### Analytic strategy

We modeled the associations between relationship processes and genetic differences using multilevel modeling using the MIXED procedure in SPSS to control for the non-independent nature of couple data. For analyses examining measures assessed at baseline, we first estimated a model that tested for a moderation by sex for each gene. If the interaction was significant, we report the simple effects for both men and women. If the interaction was not significant, we removed the interaction term from the model, as well as participant sex if it was not significantly associated with the dependent variable, and report the main effect. For all effects, we report effect size *r* (equation below) which is comparable to a correlation coefficient [see^86^].

$$r=\sqrt{\frac{{t}^{2}}{{t}^{2}+df}.}$$

For the analyses examining the trajectory of satisfaction over 3 years, we estimated growth curves in HLM7 by regressing marital satisfaction onto separate intercepts and slopes for husbands and wives in Level 1 and then entering each gene at Level 2 to account for individual differences in the intercept term, which estimated beginning marital satisfaction, and slope terms, which estimated changes in marital satisfaction. We also estimated a second model in which the time variable was recentered such that the intercept represented marital satisfaction at the end of the study (3 years after baseline).

## Supplementary Information

Supplementary Information

## Data Availability

These data cannot be deposited in a repository because participants did not consent to have their data made publicly available and because couple member data may be identifiable to each other. We will, however, make the data available upon request for such purposes as confirming study results, conducting meta-analyses, etc. A Data Access Committee, consisting of Drs. Makhanova and McNulty will assess requests for access to the data. Inquiries should be sent to Dr. McNulty at: mcnulty@psy.fsu.edu.
