# Supplementary material for: CD38 is associated with bonding-relevant cognitions and relationship satisfaction over the first 3 years of marriage
Source: Sci Rep. 2021 Feb 3;11:2965. doi: 10.1038/s41598-021-82307-z (PMC7859203; doi:10.1038/s41598-021-82307-z)
Supplement: Supplementary file 1 — Supplementary Information [file 41598_2021_82307_MOESM1_ESM.docx]

*CD38* is Associated with Bonding-Relevant Cognitions and Relationship

Satisfaction over the First Three Years of Marriage

**Supplemental Materials**

Anastasia Makhanova,^1 *^ James K. McNulty,^2^ Lisa A. Eckel,^2^
Larissa Nikonova,^2^ Jennifer A. Bartz,^3^ and Elizabeth A. D. Hammock^2^

^1^ Department of Psychological Science, University of Arkansas, 216 Memorial Hall, Fayetteville, AR 72701

^2^ Department of Psychology, Florida State University, 1107 W. Call St., Tallahassee, FL, 32306

^3^ Department of Psychology, McGill University, 2001 McGill College Ave, Montreal, QC, H3A 1G1

Correspondence to: [ammakhan@uark.edu](mailto:ammakhan@uark.edu)

Table A

*Associations between race and bonding-relevant cognitions.*

|  |  | Association with Race  (Non-Black = 0; Black = 1) | | | | | | |
| --- | --- | --- | --- | --- | --- | --- | --- | --- |
| Dependent Variable | *b* | | *SE* | *t* | *df* | *p* | *r* |  |
| Gratitude | 0.01 | | 0.10 | 0.04 | 129.64 | .972 | .003 |  |
| Trust | **-0.82** | | **0.25** | **-3.27** | **123.06** | **.001** | **.28** |  |
| Forgiveness | -0.19 | | 0.26 | -0.73 | 125.83 | .467 | .06 |  |
| Problems with Pair-bonding | **0.87** | | **0.24** | **3.56** | **137.97** | **.001** | **.29** |  |
| Marital Satisfaction | **-0.74** | | **0.25** | **-2.98** | **129.40** | **.003** | **.25** |  |

*Note.* Bolded effects are significant at *α* level of .05

Table B

*Associations between CD38 rs3796863 and bonding-relevant cognitions, controlling for race.*

|  | Association with Race  (Non-Black = 0; Black = 1) | | | | | | | Association with rs3796863  (CC = 0; AC/AA = 1) | | | | | |  |
| --- | --- | --- | --- | --- | --- | --- | --- | --- | --- | --- | --- | --- | --- | --- |
| Dependent Variable | *b* | *SE* | *t* | *df* | *p* | *r* | *b* | | *SE* | *t* | *df* | *p* | *r* | |
| Gratitude | 0.05 | 0.26 | 0.21 | 129.56 | .209 | .02 | **-0.34** | | **0.16** | **-2.11** | **112.22** | **.037** | .20 | |
| Trust | **-0.74** | **0.25** | **-2.92** | **125.17** | **.004** | **.25** | **-0.37** | | **0.16** | **-2.24** | **118.72** | **.027** | .20 | |
| Forgiveness | -0.13 | 0.26 | 0.49 | 129.35 | .623 | .04 | **-0.45** | | **0.17** | **-2.73** | **111.80** | **.007** | .25 | |
| Problems with Pair-bonding | **0.84** | **0.24** | **3.44** | **133.68** | **.001** | **.29** | 0.27 | | 0.14 | 1.91 | 98.08 | .059 | .19 | |
| Marital Satisfaction | **-0.71** | **0.25** | **-2.86** | **123.90** | **.005** | **.25** | **-0.29** | | **0.14** | **-2.14** | **86.17** | **.035** | .22 | |

*Note.* Bolded effects are significant at *α* level of .05
